# A bacterial quorum sensing signal is a potent inhibitor of *de novo* pyrimidine biosynthesis in the globally abundant *Emiliania huxleyi*

**DOI:** 10.3389/fmicb.2023.1266972

**Published:** 2023-10-06

**Authors:** Oscar Garrett, Kristen E. Whalen

**Affiliations:** Department of Biology, Haverford College, Haverford, PA, United States

**Keywords:** HHQ, *Pseudoalteromonas*, dihydroorotate dehydrogenase, phytoplankton, quorum sensing, virus-host interactions

## Abstract

Interactions between marine phytoplankton, viruses, and bacteria drive biogeochemical cycling, shape marine trophic structures, and impact global climate. Microbially produced compounds have emerged as key players in influencing eukaryotic organismal physiology, and in turn, remodel microbial community structure. This work aimed to reveal the molecular mechanism by which the bacterial quorum sensing molecule 2-heptyl-4-quinolone (HHQ), produced by the marine gammaproteobacterium *Pseudoalteromonas* spp., arrests cell division and confers protection from virus-induced mortality in the bloom-forming coccolithophore *Emiliania huxleyi*. Previous work has established alkylquinolones as inhibitors of dihydroorotate dehydrogenase (DHODH), a fundamental enzyme catalyzing the fourth step in pyrimidine biosynthesis and a potential antiviral drug target. An N-terminally truncated version of *E. huxleyi* DHODH was heterologously expressed in *E. coli*, purified, and kinetically characterized. Here, we show HHQ is a potent inhibitor (K_i_ of 2.3 nM) of *E. huxleyi* DHODH. *E. huxleyi* cells exposed to brequinar, the canonical human DHODH inhibitor, experienced immediate, yet reversible cellular arrest, an effect which mirrors HHQ-induced cellular stasis previously observed. However, brequinar treatment lacked other notable effects observed in HHQ-exposed *E. huxleyi* including significant changes in cell size, chlorophyll fluorescence, and protection from virus-induced lysis, indicating HHQ has additional as yet undiscovered physiological targets. Together, these results suggest a novel and intricate role of bacterial quorum sensing molecules in tripartite interdomain interactions in marine ecosystems, opening new avenues for exploring the role of microbial chemical signaling in algal bloom regulation and host-pathogen dynamics.

## Introduction

Interactions between heterotrophic bacteria and eukaryotic phytoplankton are fundamental to marine ecosystems (Buchan et al., 2014; Seymour et al., 2017). These ubiquitous interactions impact primary production on a global scale, driving biogeochemical cycles, shaping marine trophic structures, and influencing global climate (Azam and Malfatti, 2007; Buchan et al., 2014; Seymour et al., 2017). Bacterium-phytoplankton interactions are diverse, ranging from mutualist nutrient exchanges to predatory algicidal release, with specific pairs of bacteria and phytoplankton capable of both pathogenic and symbiotic relationships (Azam et al., 1983; Mayali and Azam, 2004; Barak-Gavish et al., 2023). Driving these dynamic interactions are excreted chemical compounds, which can modulate microbial metabolism and trigger shifts in community composition (Seyedsayamdost et al., 2011; Segev et al., 2016; Seymour et al., 2017; Barak-Gavish et al., 2023). By elucidating the eukaryotic molecular targets of bacterial compounds, we can establish critical mechanistic links between chemically mediated interactions and their ecological consequences, strengthening our ability to make accurate predictions about fundamental organismal interactions that form the basis of marine ecosystems.

The broad spectrum of compounds that influence microbial interactions, collectively termed infochemicals (Müller et al., 2020; Schmidt and Saha, 2021), orchestrate changes in microbial behavior (Schmidt and Saha, 2021). In bacterium-phytoplankton interactions, the exchange of infochemicals takes place primarily within the phycosphere, the area immediately surrounding the phytoplankton cell (Seymour et al., 2017). Quorum sensing (QS) signals, a class of diffusible infochemicals known to facilitate bacterial communication, have recently emerged as mediators of bacterium-phytoplankton interactions (Harvey et al., 2016) with direct influence on marine biogeochemical cycles (Urvoy et al., 2022). In bacterial communities, secreted QS signals induce community-wide shifts in gene expression, coordinating responses to changing environments (Ng and Bassler, 2009). It has also been demonstrated that eukaryotic hosts can “listen in” on QS signals, triggering a shift in host gene expression, often leading to an immune response (Lowery et al., 2008). QS-mediated interdomain interactions are well-established in human-microbial associations (Kariminik et al., 2017), but have only recently begun to be characterized in phytoplankton-bacterial associations (Dow, 2021).

Alkylquinolones are a class of QS molecules originally isolated from the human pathogen *Pseudomonas aeruginosa* and include both the *Pseudomonas* quinolone signal (PQS) and its precursor 2-heptyl-4-quinolone (HHQ) (Pesci et al., 1999). This class of QS molecules has a demonstrated impact on eukaryotic physiology (Reen et al., 2011) where they have been shown to modulate the human immune response (Kim et al., 2010a,b). More recently, HHQ was isolated from the marine gammaproteobacteria *Pseudoalteromonas* sp. A757 and *Pseudoalteromonas galatheae* (Harvey et al., 2016; Paulsen et al., 2020). *Pseudoalteromonas* is a globally ubiquitous genus, ranging from 0.5% to 6% of all marine bacterioplankton species worldwide (Wietz et al., 2010). Pseudoalteromonadaceae were found to have a strong association in blooms with the globally abundant coccolithophore *Emiliania huxleyi*, comprising up to 19% of prokaryotic community by the time the bloom collapses (Câmara dos Reis et al., 2023). Previous work has established that HHQ is capable of shifting the relative abundances of both bacteria and phytoplankton in natural populations, implicating this infochemical as a compound that mediates microbial population dynamics (Whalen et al., 2019). A subsequent investigation revealed that *E. huxleyi* exposure to nanomolar concentrations of HHQ could reversibly induce S-phase arrest and prevent mortality due to infection by *Emiliania huxleyi* virus (EhV) (Pollara et al., 2021), highlighting a new role for bacterial QS signals. However, the mechanistic basis for HHQ-induced cellular stasis remains unknown.

HHQ has been shown to modulate EhV infection dynamics; however, protection from viral induced mortality must coincide with exposure to the QS signal early [< 24 h post infection (hpi)] in infection and requires remodeling of host physiology (Pollara et al., 2021; Harvey et al., 2023). Moreover, this early window in EhV infection (within 8 hpi) corresponds to the time when EhV hijacks the host’s replication machinery to fuel viral gene synthesis involved in DNA replication, nucleotide metabolism, and lipid biosynthesis in parallel with repression of host nuclear and organelle-encoded genes (Ku et al., 2020). Moreover, within 24 h of HHQ exposure, *E. huxleyi* cells undergo metabolic and transcriptional changes leading to halted DNA replication, an increase in DNA lesions, and the upregulation of the DNA damage response with a notable lack of both DNA repair and apoptosis (Pollara et al., 2021). Together, these observations suggest that the cascade of physiological changes in *E. huxleyi* induced by HHQ exposure occurs early and may prevent the replication of EhVs.

Several of these physiological changes observed in HHQ-treated *E. huxleyi* may have resulted from inhibition of dihydroorotate dehydrogenase (DHODH), a key enzyme in *de novo* pyrimidine biosynthesis (Reis et al., 2017). Coincidently, inhibition of eukaryotic DHODH has been shown to trigger S-phase arrest via severe depletion of available nucleotide pools, leading to increased DNA lesions (Fairus et al., 2017). Nucleotide availability is a known limiting factor for viral burst size (Brown et al., 2006), as giant dsDNA viruses, including EhV, upregulate host *de novo* nucleotide synthesis early in the infection cycle due to their enhanced metabolic requirements (Rosenwasser et al., 2016; Vincent et al., 2021). Therefore, inhibition of *E. huxleyi* DHODH (EhDHODH) by HHQ may prevent the early acquisition of host nucleotides to fuel viral replication. In fact, several alkylquinolones have been established as *E. coli* DHODH inhibitors (Wu and Seyedsayamdost, 2017), including the structurally similar 2-heptyl-4-(1H)-quinolone N-oxide (Horwitz et al., 2022) which differs from HHQ by just a single oxygen atom.

Here, we show that HHQ is an extremely potent inhibitor of EhDHODH, exhibiting the second most potent inhibition of any DHODH recorded to date. This inhibition is attributed to competitive binding with the ubiquinone cofactor, establishing alkylquinolones as a promising reservoir for novel inhibitory agents with species specificity. Moreover, when EhDHODH was inhibited *in vivo* using the canonical human DHODH inhibitor brequinar, reversible cellular stasis was induced, mirroring the phenotype recorded with HHQ. However, not all of the physiological alterations induced by HHQ were observed in brequinar-treated *E. huxleyi.* Unexpectedly, inhibition of EhDHODH by brequinar was observed to amplify EhV201-induced mortality of *E. huxleyi*, while simultaneously significantly decreasing viral replication and markedly diminishing viral infectivity. These findings suggest DHODH inhibition contributes to, but does not fully explain, the protective effects of HHQ on *E. huxleyi* from EhV. In sum, this work provides mechanistic insight into how HHQ, a quorum sensing molecule used in bacterial communication, impacts a fundamental metabolic pathway in a global phytoplankton and presents new avenues for exploration in the molecular underpinning of HHQ-induced viral protection.

## Methods

### *E. huxleyi* cultivation

All experiments used axenic cultures of non-lith forming *E. huxleyi* CCMP2090 (from the National Center for Marine Algae and Microbiota, East Boothbay, ME). Phytoplankton cultures were grown in 0.2 μm-filtered, autoclaved natural seawater enriched f/2-Si medium (Guillard, 1975). Cultures were grown under a light:dark cycle of 14-h/10-h (80 ± 5 μmol photons m^−2^ s^−1^) at 18°C with a salinity of 35. MM and f/2 MB purity test broths and epifluorescence microscopy were used to confirm strain purity. Cultures were transferred every 6–7 days to maintain exponential growth.

### Cell enumeration

*E. huxleyi* cells were enumerated via flow cytometry (Guava; Millipore). Cell abundance was determined using species-specific parameters including forward scatter, side scatter, and red fluorescence (695/50-nm) emissions. Samples of live cells were run at 0.59 μL s^−1^ for 3 min or until 2000 events were acquired.

Total viruses in cultures were enumerated by fixation in 1% glutaraldehyde (0.2 μm-filtered) and diluted as necessary with sterile 1X phosphate buffered saline (PBS) to ensure events were less than 500 μL^−1^ on the flow cytometer. Diluted samples were stained with 1X SYBR Green (Invitrogen, Waltham, MA), incubated for 10 min at 80°C in the dark, then counted on the flow cytometer as described in (Harvey et al., 2023). Media only (f/2-Si) blanks were treated in parallel to correct for background signals.

To enumerate the concentration of viral particles actively causing cellular lysis, a most probable number assay (MPN) was used (Jarvis et al., 2010). For each treatment, equal volume aliquots of each biological replicate were pooled then serially diluted 10-fold in a stepwise fashion. A 20 μL aliquot from each dilution or undiluted pooled sample was transferred to six replicate wells of a 96-well plate containing 180 μL of axenic, exponentially growing *E. huxleyi* CCMP2090 at 1.0 × 10^6^ cells mL^−1^. The plates were incubated under general culturing conditions for 144 h before *E. huxleyi* chlorophyll fluorescence, a proxy for cell abundance, was measured via SpectraMax M2e (Molecular Devices, San Jose, CA). Wells observed to have less than or equal to half of the fluorescence of the mean of control wells were designated as “cleared,” and the MPN was calculated using the EPA MPN calculator^1^ with a Cornish & Fisher Limit of Approximation Type.

### Heterologous EhDHODH expression and purification

The sequence coding for the enzyme DHODH (UniProtID: R1EHA9) from *E. huxleyi* CCMP1516 genome was used for heterologous expression. Class 2 DHODHs previously expressed and purified often have the N-terminal transmembrane domain and mitochondrial localization region truncated, enhancing solubility in an *E. coli* expression system with minimal impact on activity (Baldwin et al., 2002; Garavito et al., 2019). For EhDHODH, an appropriate start site truncating this N-terminal region was determined by MUSCLE alignment (Edgar, 2004) in MEGA11 (v11.0.13) with previously expressed and purified DHODHs from *Solanum tuberosum* DHODH (UniProtID: M1BCR0), *Phytophthora infestans* DHODH (UniProtID: I7EMP0), *Arabidopsis thaliana* DHODH (UniProtID: P32746), *Plasmodium falciparum* DHODH (UniProtID: Q08210), and *Homo sapiens* DHODH (UniProtID: Q02127) (Supplementary Figure S1), yielding residue 21 from EhDHODH as an appropriate start site. Classification of the excluded 20-residue sequence as a mitochondrial signal peptide was performed using DeepLoc v2.0 (Thumuluri et al., 2022). The truncated gene was incorporated into the expression vector pET24a along with an N-terminal eight-residue histidine tag (Supplementary Figure S2) and the resulting plasmid was transformed into *E. coli* BL21(DE3) Star (ThermoFisher Scientific, Waltham, MA).

Protein expression, purification, and refolding were performed by GenScript (Piscataway, NJ). Briefly, ΔN20EhDHODH expression was induced in 1 L of transformed *E. coli* at OD_600_ 1.2 with 0.5 mM IPTG for 16 h at 15°C. The cells were harvested via centrifugation and lysed by sonication in lysis buffer (50 mM Tris–HCl, 500 mM NaCl, 1 mM TCEP, 0.5% TritonX-100, pH 8.0). The lysate was centrifuged to obtain pellets containing insoluble EhDHODH in inclusion bodies. The pellets were solubilized in 50 mM Tris–HCl, 7 M guanidine hydrochloride, 1 mM TCEP, pH 8.0, and the solubilized recombinant ΔN20EhDHODH was then purified using a Ni-NTA resin applied to a gravity flow column. Loosely bound proteins were washed from the column with 50 mM Tris–HCl, 8 M urea, pH 8.0, and ΔN20EhDHODH was eluted using 50 mM Tris–HCl, 8 M urea, with a series of increasing (20–300 mM) imidazole (pH 8.0) concentrations. The isolated ΔN20EhDHODH was refolded by dialysis into the final buffer containing 50 mM Tris–HCl, 150 mM NaCl, 0.5 M L-arginine, 10% glycerol, 1 mM DTT, pH 9.0. The yields of purified ΔN20EhDHODH protein were 7.65 mg/L of cell culture and the expected size for truncated recombinant ΔN20EhDHODH was 44.5 kDa. Yield and purity (≥90%) were confirmed via SDS-PAGE (4%–20% gradient) and Coomassie staining for visualization of isolated proteins (Supplementary Figure S3).

Because ΔN20EhDHODH was expressed and purified without flavin mononucleotide (FMN) supplementation, ΔN20EhDHODH was reconstituted with FMN by incubating equal volumes of 280 μg mL^−1^ EhDHODH and 37.2 μM FMN for at least 3 min on ice. The reconstituted enzyme was diluted in 50 mM Tris–HCl pH 8.0, 150 mM KCl, 0.1% Triton X-100, 10% glycerol to a 2X working stock immediately before use in downstream enzyme assays.

Protein concentration was measured using the bicinchoninic assay (Pierce, Waltham, MA) with bovine serum albumin as the standard. Absorbance was measured in 96-well plate format on a SpectraMax M2e.

### Colorimetric DHODH activity assay

In our standard assay, DHODH activity was measured by the reduction of 2,6-dichlorophenol-indophenol (DCIP) at 600 nm (ε = 19,100 M^−1^ cm^−1^) incubated at 22°C in a reaction mixture containing 50 mM Tris–HCl pH 8.0, 150 mM KCl, 0.1% Triton X-100, 10% glycerol, 1 mM L-dihydroorotate (DHO), 0.1 mM DCIP, 0.1 mM decylubiquinone (Qd) with final concentrations of 31 nM ΔN20EhDHODH or 10 nM HsDHODH (biotechne, Minneapolis, MN, Cat. No. 10062-DD-020) (Zameitat et al., 2007). To determine the kinetic constants, DHO concentration was varied from 3.1 to 1,600 μM while keeping Qd constant at 100 μM, or by varying Qd concentration from 1.2 to 600 μM with DHO fixed at 1 mM while keeping all other concentrations the same as above. Background oxidase activities due to direct reduction of DCIP by the enzyme in absence of Qd were subtracted from the activities measured in the presence of Qd (Reis et al., 2017; Garavito et al., 2019). Kinetic constants *K_m_* and *v*_max_ were calculated by fitting the data to the Michaelis–Menten equation (Eq. 1):


(1) $$v=\frac{v_{\max}\cdot \;\;\left[S\right]}{K_{m}+\left[S\right]}$$

using GraphPad Prism v9 software. The apparent *k*_cat_ was calculated by fitting the data to the equation (Eq. 2):


(2) $$v=\frac{E_{t}\;.\;k_{cat}\;.\;\left[S\right]}{K_{m}+\left[S\right]}$$

where $E_{t}$ is the concentration of catalytic sites (set to 31 nM, the concentration of enzyme used in the standard assay) using GraphPad Prism v9 software.

Stock solutions of 20 mM HHQ were prepared in DMSO and kept at −20°C until day of use, and 20 mM brequinar stock solutions were prepared in DMSO fresh on the day of use. For the dose–response experiments with DHODH inhibitors, the enzyme was incubated with concentrations of HHQ ranging from 0.001 to 100 μM, or brequinar ranging from 3 to 739 μM, or a volumetric equivalent solvent control. Activities were measured with the standard DCIP assay with saturating concentrations of Qd (100 μM) and DHO (1 mM), in the presence of the inhibitor. The final concentration of 0.5% DMSO used in the assay solutions did not impact the activity of either recombinant human or *E. huxleyi* DHODH. IC_50_ values were calculated, and 95% confidence intervals were estimated using GraphPad Prism v9 software by fitting the log transformation of the response variable (*I*; inhibitor concentration) by non-linear regression to the equation (Eq. 3):


(3) $$Y=\mathrm{Bottom}+\frac{\left(\mathrm{Top}-\mathrm{Bottom}\right)}{\left(1+10^{\left(I-{\mathrm{logIC}}_{50}*H\right)}\right)}$$

where the Hill slope (*H*) is −1 and “Top” and “Bottom” are the plateaus in initial enzymatic reaction velocity. Estimates of the *K_i_* for HHQ suggested this value would be at or below the concentration of enzyme used in our assays necessitating the use of Morrison’s equation used to determine the *K_i_* for tightly binding inhibitors (Copeland et al., 1995; Murphy, 2004). To calculate *K_i_* for HHQ, GraphPad Prism v9 was used to fit the data to the Morrison equation (Eq. 4) for tightly binding inhibitors (Copeland et al., 1995; Murphy, 2004).

(4) $$v_{i}=v_{0}\;.\;\left(1-\frac{\left(\left[E\right]+\left[I\right]+K_{i}^{\mathrm{app}}\right)-\sqrt{{\left(\left[E\right]+\left[I\right]+K_{i}^{\mathrm{app}}\right)}^{2}-4\left[E\right]\left[I\right]}}{2\left[E\right]}\right)$$.

where [*E*] is the enzyme concentration, [*I*] is the inhibitor concentration, *v_i_* is the measured initial velocity, $v_{0}$ is velocity without inhibitor, and the apparent *K_i_* (*K_i_*^app^) is related to the *K_i_*, substrate concentration [*S*], and *K_m_* for the substrate shown in Eq. 5.

(5) $$K_{i}^{\mathrm{app}}=K_{i}\left(1+\frac{\left[S\right]}{K_{m}}\right)$$.

For assessing the inhibition mechanism of HHQ, Dixon-plots were constructed by assaying the activity of ΔN20EhDHODH at 20, 40, and 80 nM HHQ while varying DHO concentrations from 12.5 to 1,600 μM or Qd concentrations from 1.17 to 150 μM (Copeland, 2000; Yoshino and Murakami, 2009). The reciprocal of the initial velocities was plotted against HHQ concentration, and a linear regression analysis was performed to construct trendlines (GraphPad Prism v9). Given the tight binding nature of HHQ, we recognize the limitations of this graphical method for determining inhibitory mechanisms (Copeland et al., 1995).

### Molecular docking

Google DeepMind’s AlphaFold v2.0 (Jumper et al., 2021) was queried with the non-truncated amino acid sequence of EhDHODH (UniProtID: R1EHA9), and the resulting structure was used as the basis for molecular docking. The substrate DHO, cofactors FMN and decylubiquinone, and putative inhibitors HHQ and brequinar were docked to the predicted structure using AutoDock Vina v1.1.2 (Trott and Olson, 2010; Eberhardt et al., 2021). The top output structures were visualized using UCSF ChimeraX v1.6.1 (Pettersen et al., 2021).

### Growth experiments

For the dose–response experiments, axenic *E. huxleyi* CCMP2090 in exponential phase at an initial concentration of 1.0 × 10^5^ cells mL^−1^ were grown in triplicate in 24-well plates exposed to nine brequinar concentrations ranging from 1 to 1,000 μM for 144 h. Plates were kept under general cultivation conditions, and cell abundance was monitored daily via flow cytometry as described above. The growth rate (μ d^−1^) over 72 h was calculated using the exponential growth equation (Eq. 6) (Harvey et al., 2016),


(6) $$\mathrm{Growth rate}=\ln\left(A_{f}/A_{i}\right)/\left(T_{f}-T_{i}\right)$$

where *A* is cell abundance at the initial and final time point (72 h), and *T* is days of growth over the first 3 days of the experiment. The growth rate was plotted against the concentration of brequinar to determine the concentration of compound resulting in 50% growth inhibition (IC_50_). The IC_50_ for brequinar was calculated using Eq. 3 as described above.

### Infection assays for viral enumeration

To investigate the impact of brequinar on EhV infection dynamics, triplicate 20 mL cultures containing exponentially growing axenic *E. huxleyi* CCMP2090 at an initial concentration of 9.0 × 10^4^ cells mL^−1^ were inoculated with EhV201 (1:1 virus:host ratio). Concurrently, cells were exposed to brequinar at a concentration shown to induce cellular stasis for 72 h (135 μM brequinar) and just below that concentration (75 μM brequinar), allowing for continued *E. huxleyi* growth. Triplicate control cultures were prepared with both viral lysate and a volumetric equivalent of DMSO. Cultures were harvested daily for *E. huxleyi* cell counts and EhV enumeration as described above. A pooled sample of all three replicates for each treatment were used to perform an MPN assay to enumerate the concentration of virus actively causing cellular lysis beginning at 24 hpi and performed every subsequent 24 h until 144 hpi.

### Statistical analyses

All statistical analyses were done using GraphPad Prism v9. Experiments conducted to examine differences in cell or viral abundances over time were tested for significance using repeated measures ANOVA with Sidak’s multiple comparison *post hoc* tests. Any *p*-values less than or equal to 0.05 were considered statistically significant in all tests.

## Results

### Expression, purification and kinetic analysis

EhDHODH can be categorized as a class 2 DHODH, as the enzyme contains both an N-terminal mitochondrial signal peptide and an N-terminal helical domain facilitating binding of a ubiquinone cofactor. Following published literature on expressing class 2 DHODHs (Baldwin et al., 2002; Zameitat et al., 2007; Garavito et al., 2019), the N-terminal mitochondrial signal peptide (first 20 residues) was truncated based on amino acid alignments with previously expressed and purified DHODHs including *S. tuberosum* DHODH, *P. infestans* DHODH, *A. thaliana* DHODH, *P. falciparum* DHODH, and *H. sapiens* DHODH (Supplementary Figure S1). To evaluate the potential inhibitory effects of HHQ on EhDHODH, ΔN20EhDHODH was heterologously expressed and purified from *E. coli* BL21. This truncation, a common method to remove the hydrophobic transmembrane anchor (Garavito et al., 2019), was insufficient to solubilize ΔN20EhDHODH in our *E. coli* expression system, necessitating purification and refolding from inclusion bodies formed during expression. SDS-PAGE confirmed the successful purification and expected molecular weight of ΔN20EhDHODH (Supplementary Figure S3).

**Figure 1:**
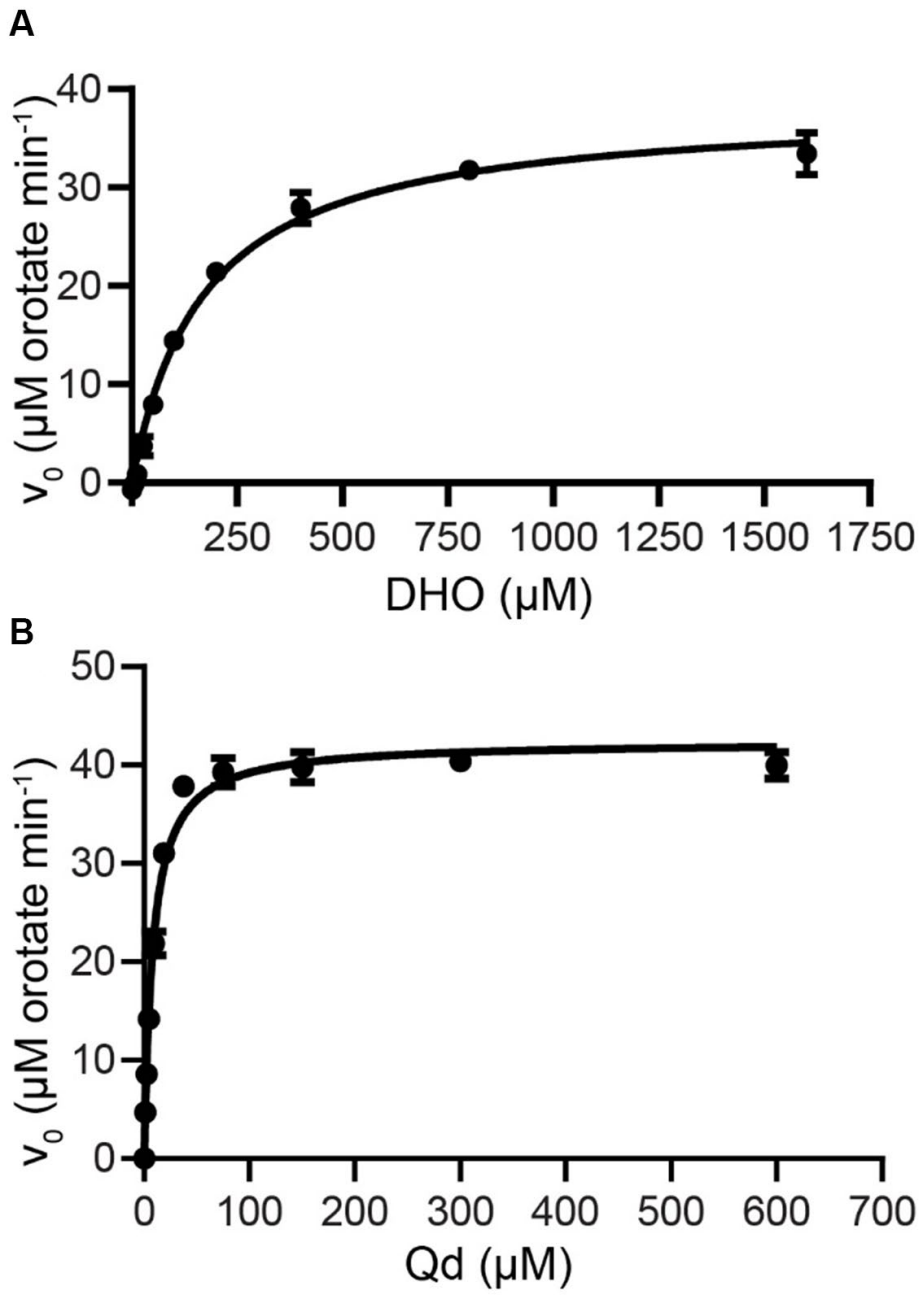
Steady-state kinetics of purified truncated recombinant *E. huxleyi* DHODH. ΔN20EhDHODH saturation curves for substrate **(A)** L-dihydroorotate (DHO) and cofactor **(B)** decylubiquinone (Qd) are shown. Non-linear regression analysis was used to fit the curves to the Michaelis–Menten equation (Eq. 1). Mean and standard deviation of triplicate experiments shown.

Saturating substrate and cofactor concentrations, as well as kinetic parameters of ΔN20EhDHODH were established and compared to other DHODHs (Figure 1, Table 1). ΔN20EhDHODH demonstrated a specific activity of 31 ± 1 μmol min^−1^ mg^−1^, a *k*_cat_^app^ of 23 ± 1 s^−1^, a *K_m_*^app^ of 170 ± 30 μM for DHO, and a *K_m_*^app^ of 8 ± 1 μM for Qd.

**Table 1:** Apparent kinetic and inhibitory parameters of *E. huxleyi* DHODH, human DHODH and their inhibitors.

Organism	*K*_*m*, DHO_ (μM)	*K*_*m*, Qd_ (μM)	*v*_max_ (μmol min^−1^ mg^−1^)	*k*_cat_ (s^−1^)	*k*_cat_/ *K*_*m*, DHO_ (×10^6^, M^−1^ s^−1^)	*k*_cat_/*K*_*m*, Qd_ (×10^6^, M^−1^ s^−1^)	IC_50, HHQ_ (μM)	IC_50, breq._ (μM)	*K*_*i*, HHQ_ (nM)	*K*_*i*, breq._ (nM)
*H. sapiens*	10	14	ND	75	7.5	5.4	NI	0.061	NI	25
*E. huxleyi*	170 ± 30	8 ± 1	31 ± 1	23 ± 1	0.14 ± 0.03	2.8 ± 0.6	0.040 ± 0.008	43 ± 11	2.3 ± 0.7	ND

NI, not inhibited; ND, not determined. Human DHODH *K*_*m*, DHO_, *K*_*m*, Qd_ and *k*_cat_ were obtained from Ullrich et al. (2001), and *K*_*i*, breq._ was obtained from Cuthbertson et al. (2020). Enzyme activities were measured using the colorimetric assay, at 22°C, pH 8.0, in 50 mM Tris–HCl, 150 mM KCl, 10% glycerol, 0.1% Triton X-100, and 100 μM DCIP. DHO was fixed at 1 mM with variable Qd concentrations, and Qd was fixed at 100 μM with variable DHO concentrations.

### HHQ is a potent inhibitor of EhDHODH

**Figure 2:**
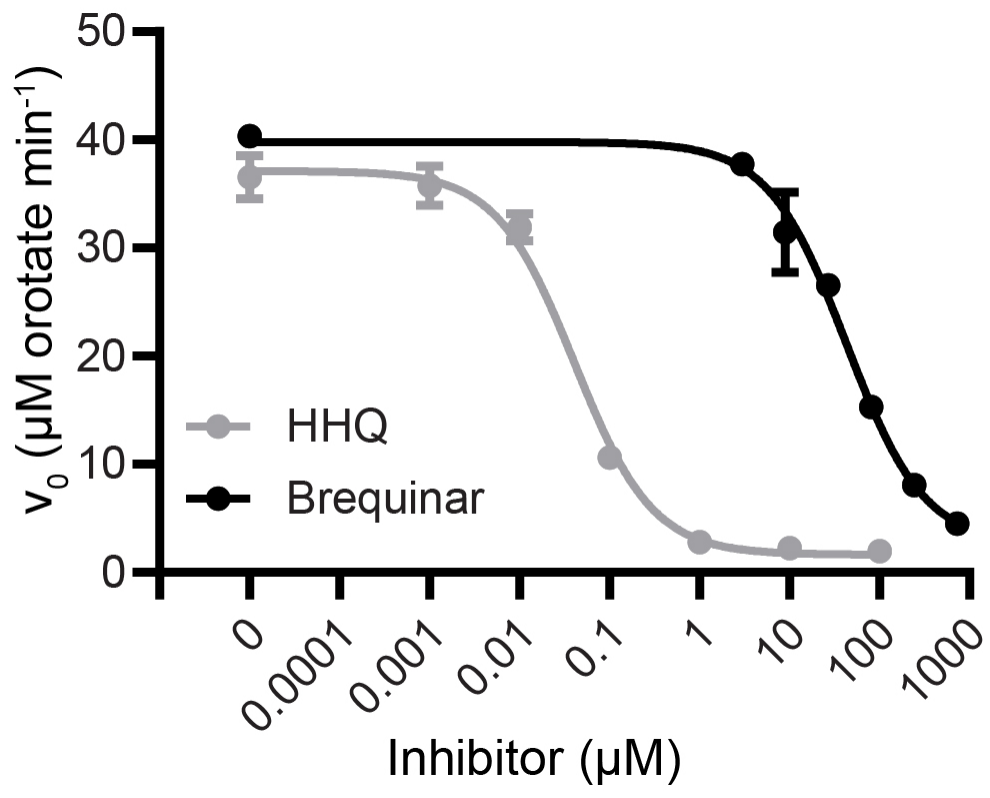
Dose–response inhibition curves of HHQ and brequinar for truncated recombinant *E. huxleyi* DHODH. Activities were measured with the standard colorimetric DHODH activity assay with saturating concentrations of Qd (100 μM) and DHO (1 mM) in the presence of 0–100 μM HHQ or 0–729 μM brequinar for ΔN20EhDHODH. Non-linear regression analysis was used to generate the trendline. Mean and standard deviation of triplicate experiments shown.

Using saturating concentrations of DHO and Qd (1 mM and 100 μM, respectively), we constructed a dose–response curve to determine the IC_50_ of HHQ (Figure 2). HHQ exhibits potent inhibition of ΔN20EhDHODH, with an IC_50_ of 40 nM against 31 nM of ΔN20EhDHODH. Estimates of the *K_i_* of HHQ suggested this value would be at or below the concentration of enzyme used in our assays necessitating the use of Morrison’s equation for tightly binding inhibitors to determine the *K_i_* (Copeland et al., 1995; Murphy, 2004). Fitting the dose–response data to the Morrison’s equation yielded a *K_i_* of 2.3 ± 0.7 nM (Table 1). For comparison, the inhibitory effect of brequinar, the canonical inhibitor of human DHODH, was substantially less potent against ΔN20EhDHODH, with an IC_50_ of 43 μM (Figure 2), a thousand-fold higher than the IC_50_ for HHQ. The inhibitory effect of HHQ was also tested against human DHODH, and no measurable dose-dependent inhibitory effect was observed up to 100 μM HHQ (data not shown). A summary of the inhibitor constants and their counterparts against human DHODH are summarized in Table 1.

To further understand the interaction between EhDHODH and HHQ, Dixon plots were constructed (Supplementary Figure S4), which indicated HHQ demonstrates competitive inhibition with respect to Qd and uncompetitive inhibition against DHO. Molecular docking of HHQ and brequinar to an AlphaFold 2.0-predicted 3D structure of EhDHODH supports the model whereby HHQ and brequinar bind to the decylubiquinone binding site (Supplementary Figure S5). In the docked structure, HHQ slots into the ubiquinone access tunnel with its alkyl chain pointing inward toward the catalytic center of the enzyme, and HHQ forms a hydrogen bond between the carbonyl of HHQ and S63. The benzene ring of HHQ is positioned at the entrance of the ubiquinone access tunnel which is comprised primarily of hydrophobic residues. Mid-way through the tunnel, the pyridine ring lines up with the charged / polar residues including S63. At the end of the tunnel, the hydrophobic alkyl chain of HHQ associates with more hydrophobic residues toward the catalytic center.

### DHODH inhibition drives HHQ-induced cellular stasis

**Figure 3:**
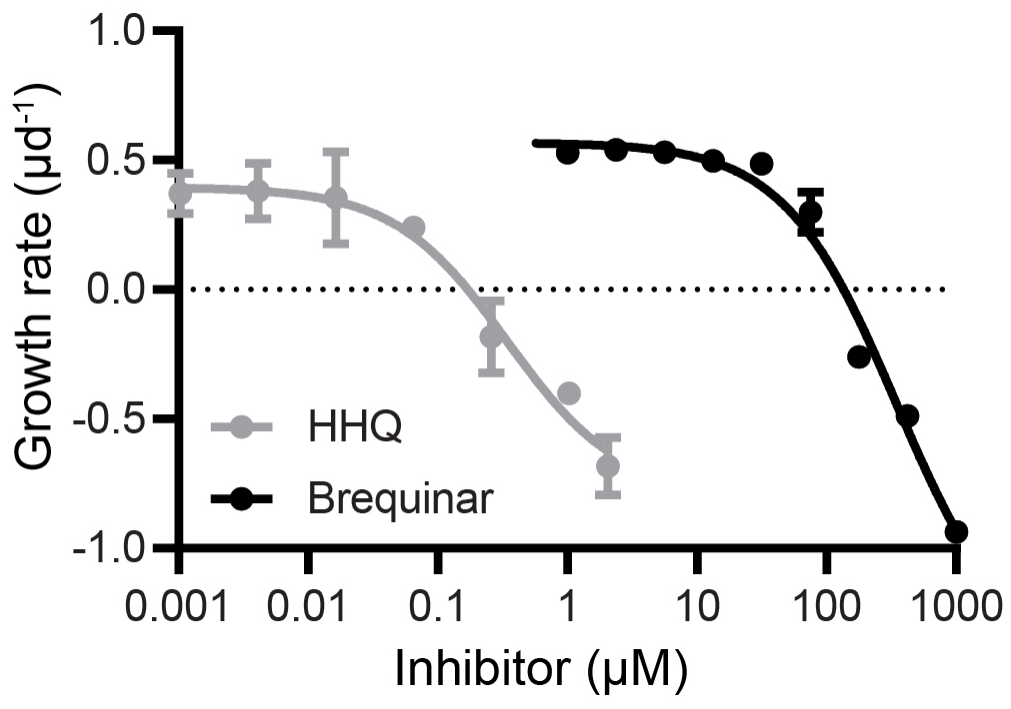
Dose–response comparison of *E. huxleyi* growth rate during exposure to HHQ and brequinar. Exponentially growing axenic *E. huxleyi* (CCMP2090) were dosed with brequinar (0–1,000 μM) or HHQ (0–0.54 μM) previously [HHQ data from Pollara et al. (2021)], then monitored for 72 h. The growth rate was calculated over 72 h and the mean and standard deviation of biological triplicates are shown.

To mechanistically connect enzymatic DHODH inhibition established by our *in vitro* studies and the cellular stasis characteristic of *in vivo* HHQ exposure (Pollara et al., 2021) we conducted a dose–response experiment with *E. huxleyi* exposed to brequinar concentrations ranging from 0 to 1,000 μM. Cellular stasis was induced at 135 μM brequinar with little to no growth or cell death over 72 h (Figure 3). For axenic *E. huxleyi* (CCMP2090), the IC_50_ of brequinar was determined to be 345 μM, while HHQ was nearly a thousand-fold lower at 363 nM (Pollara et al., 2021). To examine whether the growth inhibitory effect of brequinar was reversible, *E. huxleyi* cultures exposed to brequinar for 120-h were diluted 20-fold in fresh f/2-Si media to yield a final concentration of 6.75 μM brequinar, a concentration determined not to exhibit any inhibitory effect on *E. huxleyi* growth (Figure 3). Under these conditions, exponential growth was restored by 72 h post-dilution (Supplementary Figure S6). Unlike HHQ (Pollara et al., 2021), brequinar did not induce an increase in *E. huxleyi* cell size or chlorophyll fluorescence (Supplementary Figure S7).

### Brequinar enhances cell death in response to viral infection, but limits EhV infectivity

**Figure 4:**
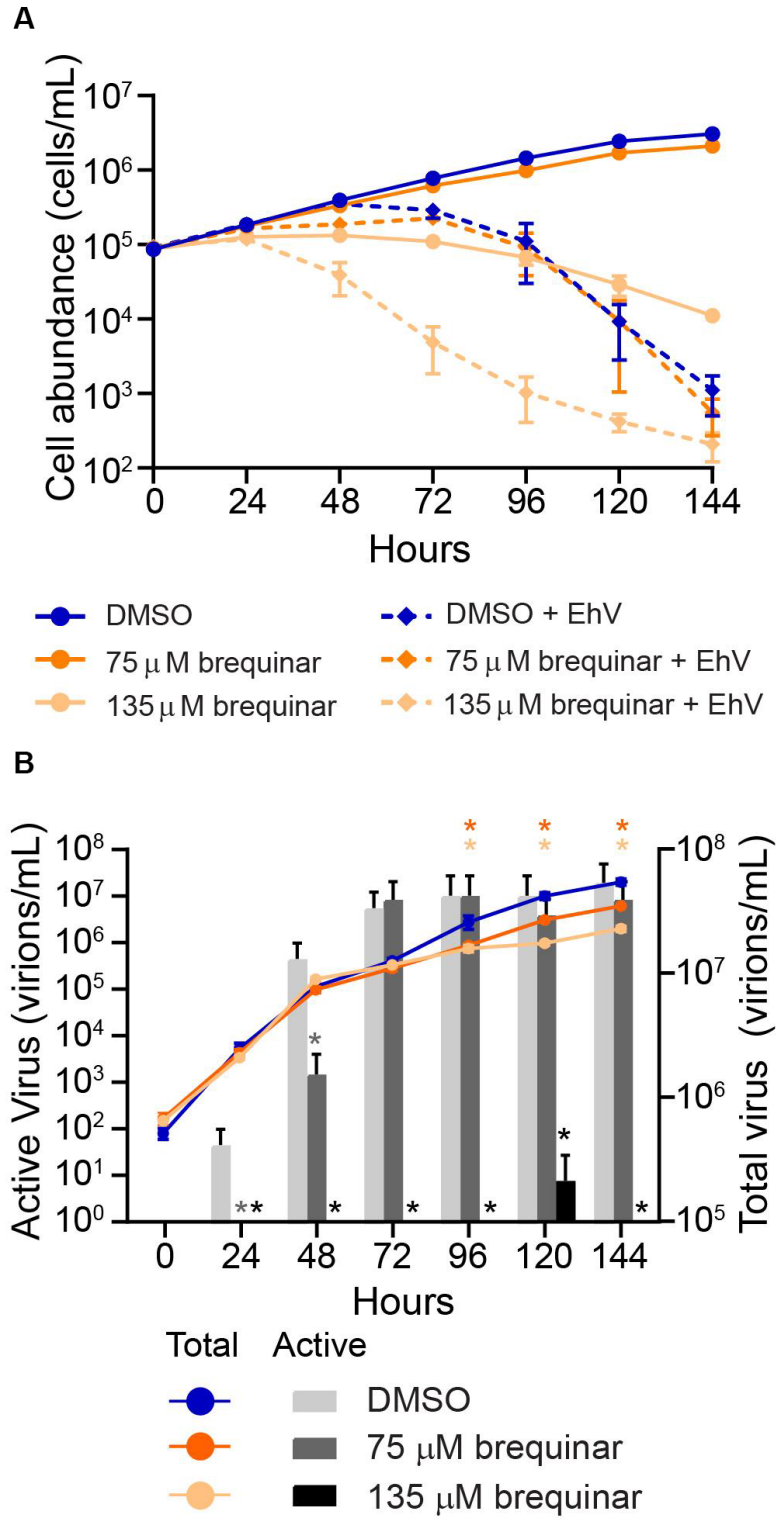
Effect of brequinar on *E. huxleyi* growth rate and viral dynamics. **(A)** *E. huxleyi* (CCMP2090) cultures were dosed with brequinar with and without *E. huxleyi* virus (EhV) 201 (1:1 host:virus ratio). *E. huxleyi* cell abundance was measured every 24 h over 144 hpi. **(B)** Infectious viral titer (left Y-axis) and total viral abundance (right Y-axis) were measured over 144 hpi. In both **(A,B)**, cell abundance and total viral abundance are shown as the mean and standard deviation of triplicates. Total virion abundance between brequinar treatments and DMSO controls were significantly different (*p*-value ≤ 0.0001, denoted with an asterisk with colors corresponding to respective brequinar treatment) at 96, 120, and 144 hpi using repeated measures ANOVA and Sidak’s *post hoc* test for significance. Infectious or active viral particles were quantified using the MPN assay from 24 to 144 hpi, and the mean and 95% confidence interval are shown. Cultures dosed with 75 μM brequinar had significantly reduced (*p*-value ≤ 0.05, denoted with an asterisk in dark grey) infectious virion abundance at 24 and 48 hpi relative to DMSO controls. Cultures dosed with 135 μM brequinar had significantly reduced (*p*-value ≤ 0.05, denoted with an asterisk in black) infectious virion abundance relative to DMSO controls at all timepoints measured.

To test whether DHODH inhibition alone was contributing to cellular protection from virus-induced mortality, *E. huxleyi* cultures were exposed to 75 or 135 μM brequinar, then infected with EhV201 at a 1:1 host-to-virus ratio. Brequinar did not protect *E. huxleyi* from virus-induced mortality; rather, cell lysis was enhanced by brequinar in infected cultures (Figure 4A). Beginning at 96 hpi, total viral abundance in brequinar-exposed infected cultures was significantly (*p*-value ≤ 0.0001) less relative to infected cultures with no brequinar addition (Figure 4B), indicating viral replication was impacted. Moreover, at 135 μM brequinar, the concentration of virus actively causing cellular lysis was zero at all time points examined compared to control, with the exception of 120 hpi (Figure 4B). At a concentration of 75 μM brequinar, a concentration which still allowed for *E. huxleyi* growth, a significant (*p*-value ≤ 0.05) decrease in infectious viral particles was observed for 24 hpi and 48 hpi compared to control (Figure 4B).

## Discussion

Previous work established that the alkylquinolone quorum sensing signal HHQ has significant effects on modulating growth and viral infection dynamics in the globally abundant coccolithophore *E. huxleyi* (Harvey et al., 2016; Pollara et al., 2021; Harvey et al., 2023), but the mechanistic basis of these effects was unknown. Alkylquinolones have been shown to inhibit *E. coli* DHODH (Wu and Seyedsayamdost, 2017; Horwitz et al., 2022), a fundamental enzyme in *de novo* pyrimidine synthesis. Moreover, the lack of available nucleotide pools is known to induce S-phase arrest in eukaryotic cells (Fairus et al., 2017), which could explain the cellular stasis previously observed in HHQ-treated *E. huxleyi* (Harvey et al., 2016; Pollara et al., 2021). Having a sufficient quantity of host-supplied nucleotides is also of critical importance during viral infection and propagation, and the resulting loss of pyrimidine production by DHODH inhibition could explain the decrease in viral abundance observed in EhV-infected *E. huxleyi* cultures treated with HHQ (Harvey et al., 2023).

Here, we establish HHQ to be a potent inhibitor of recombinant EhDHODH with a *K_i_* of 2.3 nM, placing this inhibitor second only to 2-biphenyl-4-yl-3-methyl-quinoline-4-carboxylic acid (Batt et al., 1995) as the most potent inhibitor of any DHODH reported on BindingDB (Liu et al., 2007). There has been significant interest in developing synthetic inhibitors for human and human pathogen DHODHs. These inhibitors include brequinar derivatives (Batt et al., 1995; Madak et al., 2018), leflunomide derivatives (Davies et al., 2009), and pyrazoles (Haque et al., 2002), the most potent of which having inhibitory constants ranging from 4.0 to 1.7 nM. HHQ represents the first naturally produced compound to have an inhibitory constant for a eukaryotic DHODH with potency comparable to those inhibitors developed for therapeutic use in humans.

This study provides evidence of the structural basis for HHQ’s potent and species-specific inhibition. Similar to other alkylquinolones (Wu and Seyedsayamdost, 2017; Horwitz et al., 2022), Dixon plots and *in silico* modeling both provide evidence that HHQ likely binds the ubiquinone binding site (Supplementary Figures S4, S5), thus preventing the ubiquinone-mediated regeneration of oxidized FMN (Fagan et al., 2006). DHODH inhibitors that competitively bind to the ubiquinone binding site are often species specific due to high variability in the N-terminal region of DHODH, which contains residues that line the ubiquinone channel (Hurt et al., 2006; Garavito et al., 2019). Consequently, the degree to which inhibitor structural motifs carry over between distantly related DHODHs is often limited. Indeed, HHQ’s inhibitory qualities do not extend to human DHODH. Likewise, brequinar is 1,000-fold less potent of an inhibitor for EhDHODH compared to HHQ. Previous studies found that brequinar derivatives with enhanced affinity for human DHODH formed new electrostatic interactions, including strategically positioned hydrogen-bonds within the ubiquinone channel (Madak et al., 2018). Docking results indicate a unique hydrogen bond between the carbonyl on HHQ with the hydroxyl on S63, which may stabilize HHQ in the ubiquinone channel and account for HHQ’s high affinity specific for EhDHODH.

Furthermore, when comparing the sequence of EhDHODH with DHODHs from human and the two algal species *Phaeodactylum tricornutum* and *Dunaliella tertiolecta,* shown previously to be insensitive to HHQ (Harvey et al., 2016), there is substantial variation in the residues forming the EhDHODH ubiquinone channel (Supplementary Figure S8). In docking studies with EhDHODH, only one (L36) of the 15 residues shown to be within 5 Å of decylubiquinone, HHQ, or brequinar is conserved with HsDHODH, while two residues (V14 and L47) are conserved with *P. tricornutum*, and two residues (L47 and L59) are conserved with *D. tertiolecta* (Supplementary Figure S8). Likewise, residues lining the channel within human DHODH (R136, Y356, V134, V143, and H55), deemed crucial for the binding of brequinar (Davis and Copeland, 1997; Hurt et al., 2006; Madak et al., 2018), are not conserved in the protein alignment with *E. huxleyi*, likely explaining the reduced potency of brequinar observed against EhDHODH. Together, these data indicate that HHQ inhibition of DHODH is highly species specific, likely resulting from the unique structure of EhDHODH’s ubiquinone channel.

Exposure of *E. huxleyi* cells to brequinar was used to estimate the influence of DHODH inhibition on host cell division and viral propagation dynamics. While brequinar did induce cellular stasis in *E. huxleyi,* albeit at micromolar concentrations, this inhibitor failed to mimic all of HHQ’s cellular effects, notably the increased cell size and chlorophyll fluorescence seen with HHQ treatment (Pollara et al., 2021) (Supplementary Figure S7). Following brequinar exposure at 135 μM, a decline in cell abundance was observed after 72 h in contrast to complete cellular stasis recorded for 504 h under HHQ (410 nM) treatment (Pollara et al., 2021). The loss of cells after 72 h of cellular stasis following brequinar exposure parallels studies showing DHODH inhibition eventually leading to programmed cell death (PCD) (Arnould et al., 2017; Fairus et al., 2017), which is in stark contrast to HHQ-exposed *E. huxleyi* cells, which exhibit no physiological hallmarks of PCD (Pollara et al., 2021). This raises the possibility that HHQ may interfere with additional, yet unidentified, cellular stress signaling pathway(s), which might otherwise lead to PCD.

The application of brequinar to infected cells was insufficient to protect cells from virus-induced mortality. At 75 μM brequinar, a concentration that allows for *E. huxleyi* growth, infected cultures tracked with paired controls, with no inhibition or enhancement of viral lysis observed. At 135 μM brequinar, a cellular stasis-inducing concentration analogous to 410 nM HHQ treatment used previously (Pollara et al., 2021), *E. huxleyi* cell death in response to EhV infection was enhanced. This result was unexpected due to the wealth of literature reporting DHODH inhibitors as promising antivirals, including brequinar (Qing et al., 2010; Hoffmann et al., 2011; Wang et al., 2011; Lucas-Hourani et al., 2013; Luthra et al., 2018; Demarest et al., 2022; Zheng et al., 2022). Our observations prompt questions of whether the increased cell death is a result of DHODH inhibition in *E. huxleyi* or non-DHODH-related cytotoxic effects of brequinar that synergize with the stress of viral infection. In human cell lines, the dose of brequinar capable of decreasing dengue viral infectivity in human cell lines ranged from 0.1 to 3 μM (Qing et al., 2010), corresponding to 33- to 1,000-fold below the cytotoxicity threshold concentration (approximately 100 μM) following 24 h of drug exposure (Hoffmann et al., 2011). Because brequinar is a poor inhibitor of EhDHODH, the concentration that would likely inhibit viral lysis in *E. huxleyi* cells overlaps with the cytotoxicity threshold of brequinar. It is therefore difficult to resolve whether the enhanced lysis during infection was due to DHODH inhibition or cytotoxic effects of brequinar.

Though lysis was not prevented by brequinar in infected cultures, there were significant changes in total virion and infectious virion production over the course of infection. Both concentrations of brequinar tested resulted in a significant decrease in total virion abundance relative to infected controls starting at 96 hpi (Figure 4B). However, the brequinar treatments reached a maximum total virion abundance comparable to the infected controls, while HHQ treatment resulted in a 10-fold decrease in total virion abundance relative to the control (Harvey et al., 2023), suggesting HHQ is more effective than brequinar at preventing viral DNA replication. In HHQ-exposed cells, infectious virion production saw a 1,000-fold reduction compared to controls (Harvey et al., 2023). Similarly, 135 μM brequinar treatment showed complete elimination of infectious virion production with the exception of 120 hpi. These findings suggest that DHODH inhibition contributes to a decrease in both total and infectious virion production and may largely act on post-viral DNA replication processes.

DHODH inhibitors have previously been shown to limit the titer of infectious negative-sense RNA viruses, positive-sense RNA viruses, DNA viruses, and retroviruses (Qing et al., 2010; Hoffmann et al., 2011; Wang et al., 2011; Lucas-Hourani et al., 2013; Luthra et al., 2018; Demarest et al., 2022; Zheng et al., 2022). This broad-spectrum antiviral activity is thought to have resulted from insufficient nucleotide pools available for viral replication (Hoffmann et al., 2011; Wang et al., 2011; Luthra et al., 2018; Demarest et al., 2022). However, DHODH inhibition also appears to activate innate cellular immunity, including processes regulated by the interferon regulatory factor-1 (IRF1), a transcription factor known to maintain an “antiviral state” in mammalian cells (Lucas-Hourani et al., 2013; Luthra et al., 2018; Panda et al., 2019). It is therefore possible that DHODH inhibition in algal cells triggers an analogous antiviral pathway(s) such as induction of a hypersensitive PCD response (Heath, 2000; Bidle, 2016), downregulation of viral lipid biosynthesis (Rosenwasser et al., 2014), or RNAi-mediated gene silencing (De Riso et al., 2009).

Adding additional complexity are reports of HHQ suppressing human immune responses by inhibition of macrophage activation and nitric oxide (NO) production (Kim et al., 2010b), as well as suppression of innate immunity by the inhibition of the transcription factor NF-κB and its downstream target genes (Kim et al., 2010a). Parallels can be drawn between mammalian and *E. huxleyi* cells in their response to HHQ. Recent findings have shown that *E. huxleyi* exposure to HHQ limits production of NO during viral infection (Harvey et al., 2023), which is well established to play a role in regulating innate cellular immunity in plants (Yoshioka et al., 2011). Indeed, co-induction of NO and reactive oxygen species (ROS) leads to a hypersensitive PCD immune response in plants (Delledonne et al., 2001), and in phytoplankton both NO and ROS are considered hallmarks of PCD in general (Bidle, 2016), though their specific roles in regulating innate immunity have yet to be explored.

Though suppression of innate immunity by HHQ at first seems contradictory to viral protection, when we consider the role of PCD in EhV infection, the utility of this suppression becomes evident. During EhV infection, the virus tightly controls host PCD activation pathways to both prevent the host from activating PCD prematurely as a strategy to rid itself of the virus and facilitate viral release later in the infection cycle (Bidle and Falkowski, 2004). EhV infection coincides with NO and ROS production (Evans et al., 2006; Schieler et al., 2019), as well as induction of an autophagy-like process (Schatz et al., 2014), each of which are typically referred to in the context of immune responses in mammalian and plant systems, but here are hijacked by the virus and are critical for completion of the EhV infection cycle (Schatz et al., 2014; Sheyn et al., 2016; Schieler et al., 2019). Therefore, HHQ-mediated suppression of these “immune” responses could cripple EhV’s ability to complete its infection and propagation cycle. In combination with potent DHODH inhibition limiting the availability of nucleotide pools, HHQ likely has additional complementary pathways that lead to reduction of both viral DNA replication and viral-mediated host lysis.

A possible secondary target of HHQ is poly (ADP-ribose) polymerase (PARP), a master regulator of the DNA damage response (DDR) (Jagtap and Szabó, 2005). The accumulation of DNA lesions characteristic of HHQ exposure (Pollara et al., 2021), and DHODH more broadly, would ordinarily overstimulate the PARP-regulated DDR, depleting ATP and NAD+ pools, thereby initiating apoptotic pathways (Yu et al., 2002). Moreover, PARP has also been extensively implicated in innate cellular immunity of both mammalian and plant cells (Chang and Alvarez-Gonzalez, 2001; Hassa et al., 2003; Adams-Phillips et al., 2010). Expression of genes regulated by NF-κB are known to be dependent on PARP1 activity (Chang and Alvarez-Gonzalez, 2001; Hassa et al., 2003), and inhibition of PARP in *Arabidopsis* has been shown to inhibit plant immune responses downstream of pathogen-induced ROS production (Adams-Phillips et al., 2010). Furthermore, benzimidazole derivatives, which share an internal structural motif with HHQ, are known dual inhibitors of DHODH and PARP (Abdullah et al., 2015), and HHQ has been reported to inhibit human PARP at a concentration of 50 μM (Pollara et al., 2021). It would therefore be of interest to determine if HHQ could inhibit *E. huxleyi* PARP, thereby averting the PCD response that would typically be realized by DHODH inhibition and / or viral infection.

Fundamental questions remain about the ecological impact of HHQ-mediated DHODH inhibition. What incentive is there for *Pseudoalteromonas* spp. to inhibit *de novo* pyrimidine synthesis in *E. huxleyi*, thereby inducing cellular stasis? Accumulation of DNA lesions, such as those characteristic of HHQ-exposure and DHODH inhibition in general, causes a remodeling of cellular metabolism that results in an increase in ATP production to fuel DNA repair (Milanese et al., 2019). When DNA repair is faulty, this ATP accumulates and in turn inhibits glycolysis, which is thought to prevent superfluous ATP production (Milanese et al., 2019). In line with this metabolic remodeling, previous work established that *E. huxleyi* exposure to HHQ results in an increased transcript abundance of enzymes associated with the tricarboxylic acid cycle, which would correlate with production of reducing equivalents for ATP production, as well as reduced transcript abundance of hexokinase, the first step in glycolysis (Pollara et al., 2021). Together these findings provide important supporting evidence for our hypothesis that an HHQ-exposed *E. huxleyi* cell converts to a fuel cell, incapable of cell division yet producing a surplus of energy storage molecules, all the while preserving glucose reserves. It is conceivable that such a metabolic profile shift could benefit bacterial growth and may be preferable to bacterially-mediated predation of the phytoplankton cell. However, metabolomic characterization of HHQ-induced changes in metabolic exchange between the algal host and bacterium are needed to validate this hypothesis.

The discovery that a bacterial communication molecule, HHQ, is a potent inhibitor of a fundamental enzyme in eukaryotic nucleotide synthesis establishes a novel avenue through which microbial chemical communication can shape algal physiology and, in turn, bloom dynamics and microbial communities more broadly. The high affinity and specificity of HHQ for EhDHODH suggests an intimate co-evolutionary relationship between the host and bacterium. Future work elucidating how HHQ might alter the chemical currency between *Pseudoalteromonas* spp. and its algal host *E. huxleyi* may undercover a species-specific metabolic incentive for the bacterium to induce cellular stasis in the algal host, even during viral assault. The evolution of this HHQ-mediated tripartite interdomain interaction remains elusive but unraveling the mechanistic underpinnings will undoubtably reveal a novel three-way interaction between a phytoplankton host, a bacterium, and a virus.

## Data availability statement

The data presented in this study are deposited in the STRENDA database, https://www.beilstein-strenda-db.org/, accession number V4LL20.

## Author contributions

OG: Conceptualization, Data curation, Formal analysis, Funding acquisition, Methodology, Writing – original draft, Writing – review & editing. KW: Conceptualization, Funding acquisition, Project administration, Resources, Supervision, Writing – original draft, Writing – review & editing.

## Footnotes

^1^ https://mostprobablenumbercalculator.epa.gov/mpnForm
