# Supplementary material for: A bacterial quorum sensing signal is a potent inhibitor of de novo pyrimidine biosynthesis in the globally abundant Emiliania huxleyi
Source: Front Microbiol. 2023 Oct 6;14:1266972. doi: 10.3389/fmicb.2023.1266972 (PMC10587436; doi:10.3389/fmicb.2023.1266972)
Supplement: Supplementary file 1 [file Data_Sheet_1.PDF]

Supplementary Material

A Bacterial Quorum Sensing Signal is a Potent Inhibitor of *de novo* Pyrimidine Biosynthesis in the Globally Abundant *Emiliania huxleyi*

Oscar Garrett[1], Kristen E. Whalen[1*]

*** Correspondence:** Kristen Whalen: kwhalen1@haverford.edu

[1]Department of Biology, Haverford College, Haverford, Pennsylvania, USA

Supplemental Figures

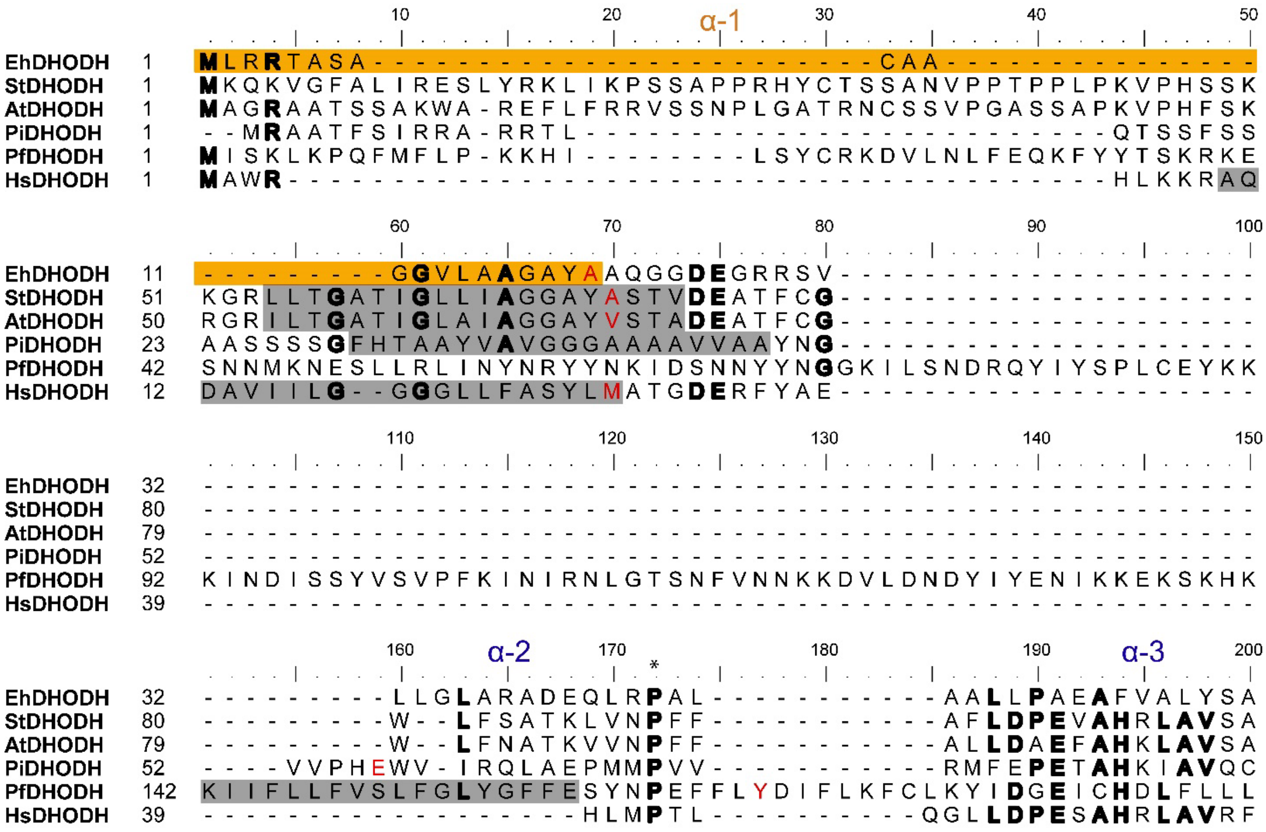

Supplemental Figure 1. Multiple sequence alignment of expressed and purified DHODHs used to determine *E. huxleyi* DHODH truncation for recombinant expression. Residues with at least 55% conservation are bolded, and those with 100% conservation are indicated with an asterisk. Highlighted in grey are transmembrane domains predicted by PredictProtein (Bernhofer et al., 2021). Residues colored red indicate reported start sites from Garavito et al., 2019 (St-, At-, and PiDHODH), and Baldwin et al., 2002 (Pf- and HsDHODH). EhDHODH = *Emiliania huxleyi*, R1EHA9, StDHODH = *Solanum tuberosum*, M1BCR0, AtDHODH = *Arabidopsis thaliana*, P32746, PiDHODH = *Phytophthora infestans*, I7EMP0, PfDHODH = *Plasmodium falciparum*, Q08210, HsDHODH = *Homo sapiens*, Q02127. The position of the first three alpha helices in EhDHODH are denoted above the alignment.

N_delta_20_EhDHODH_pET24a (6552 bp)

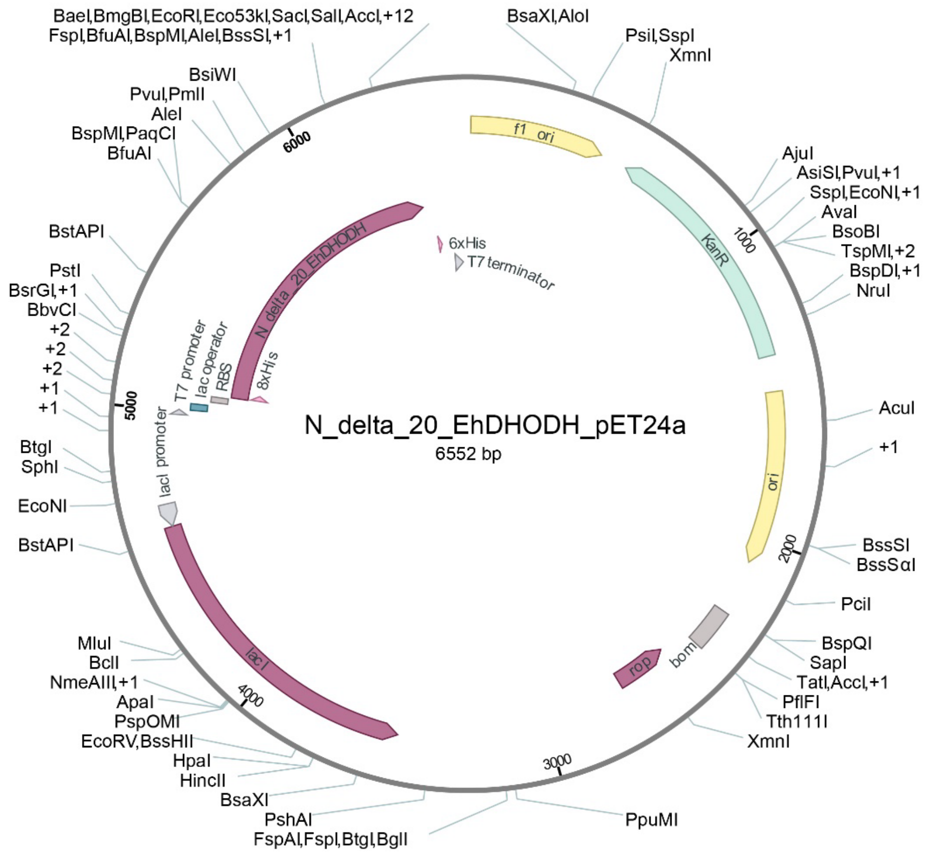

Supplemental Figure 2. Plasmid map of the pET-24a vector containing a histidine tagged, N-terminally truncated (20 residues) EhDHODH. The plasmid includes a kanamycin resistance gene, and a *lacI* promoter for induction using IPTG.

SDS-PAGE:

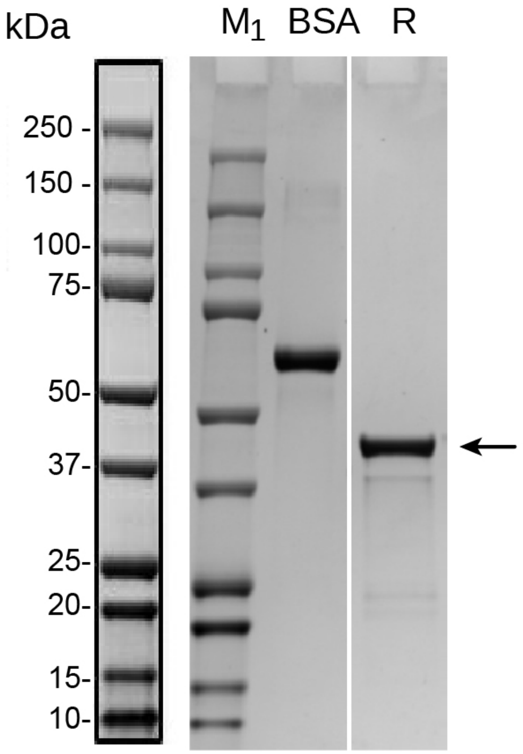

Lane M_1: Protein Marker, Bio-rad, Cat. No. 1610374S
BSA: 2.00 µg
R: Reducing condition

Supplemental Figure 3. SDS-PAGE of purified truncated recombinant EhDHODH. M_1 shows the protein marker with a guide to molecular weight to the left. A total of 2.00 µg of BSA was run as a control alongside EhDHODH in lane R. Arrow indicates ΔN20EhDHODH at the expected molecular weight near 44.5 kDa. Purity was determined to be ≥90%.

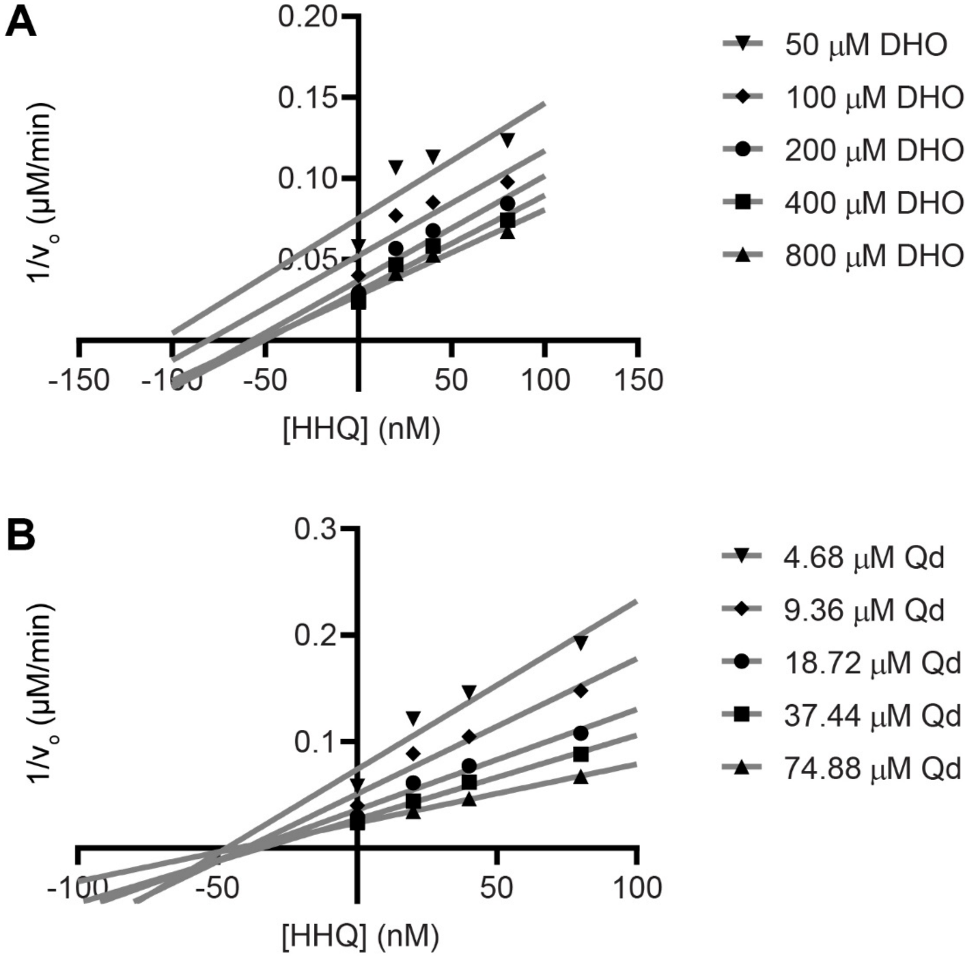

Supplemental Figure 4. Dixon plots for HHQ inhibition of purified truncated recombinant *E. huxleyi* DHODH. Concentrations of (**A**) DHO or (**B**) Qd were varied when exposed to 0, 20, 40, or 80 nM of HHQ. Plotted are the reciprocal of the mean of initial velocities. Shown are the means of triplicates.

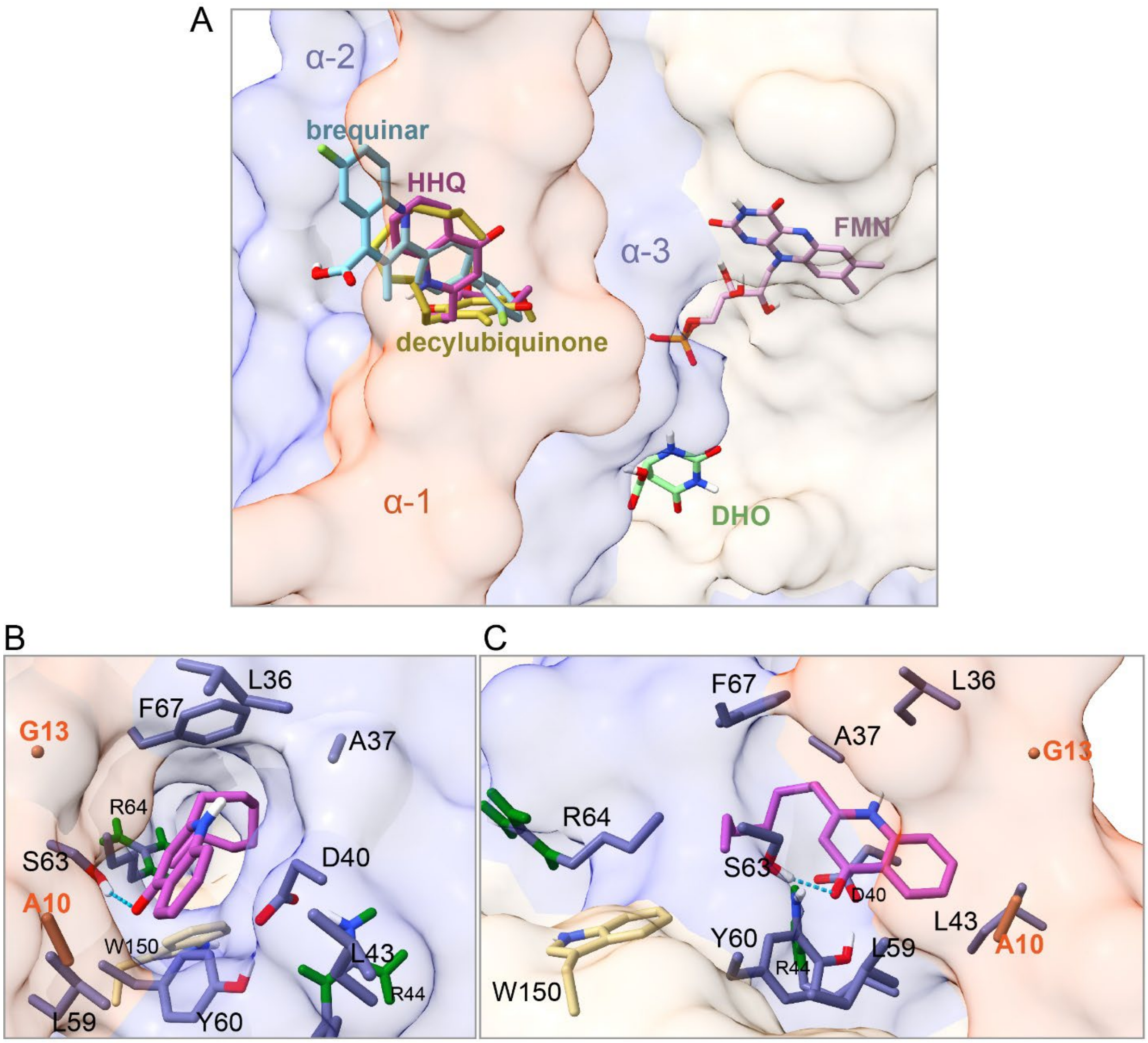

Supplemental Figure 5. ***In silico*** **model of the ubiquinone-binding pocket predicted for EhDHODH.** HHQ (magenta), brequinar (blue), and decylubiquinone (yellow) were docked to an AlphaFold 2.0 (Jumper et al., 2021) predicted EhDHODH structure using AutoDock Vina (Eberhardt et al., 2021; Trott & Olson, 2010). Also docked are FMN (pink) and DHO (green). (**A**) Depicts all docked ligands, with the docked structures of HHQ, brequinar, and decylubiquinone overlapping in the ubiquinone access tunnel. (**B**) and (**C**) show different orientations of the positioning of HHQ with its alkyl chain pointing inward toward the catalytic center of the enzyme and benzene ring positioned at the entrance of the ubiquinone access tunnel. Amino acids within 5 Å of the ligand are shown. Hydrogen bonds are depicted as blue dashes. Surfaces contributing to the ubiquinone-binding pocket made up of residues 1-20 within alpha helix 1 that were truncated in ΔN20EhDHODH are coloured orange. All other alpha helices have surfaces and residues colored in blue. The visualization was generated using UCSF ChimeraX (Pettersen et al., 2021).

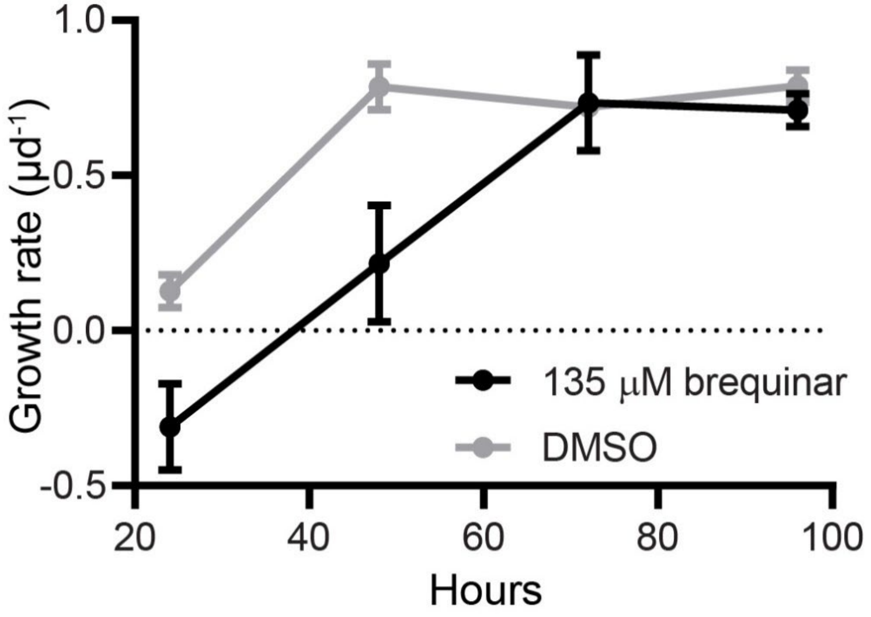

Supplemental Figure 6. Recovery of brequinar-treated *E. huxleyi* cultures over 96 h. Following exposure to 135 μM brequinar for 120 h (see Figure 4A), cultures were diluted 20-fold in fresh f/2 media and cell abundance was monitored for an additional 96 h. The growth rate (Eq. 6) was calculated over 24 h and the mean and standard deviation of biological triplicates are shown.

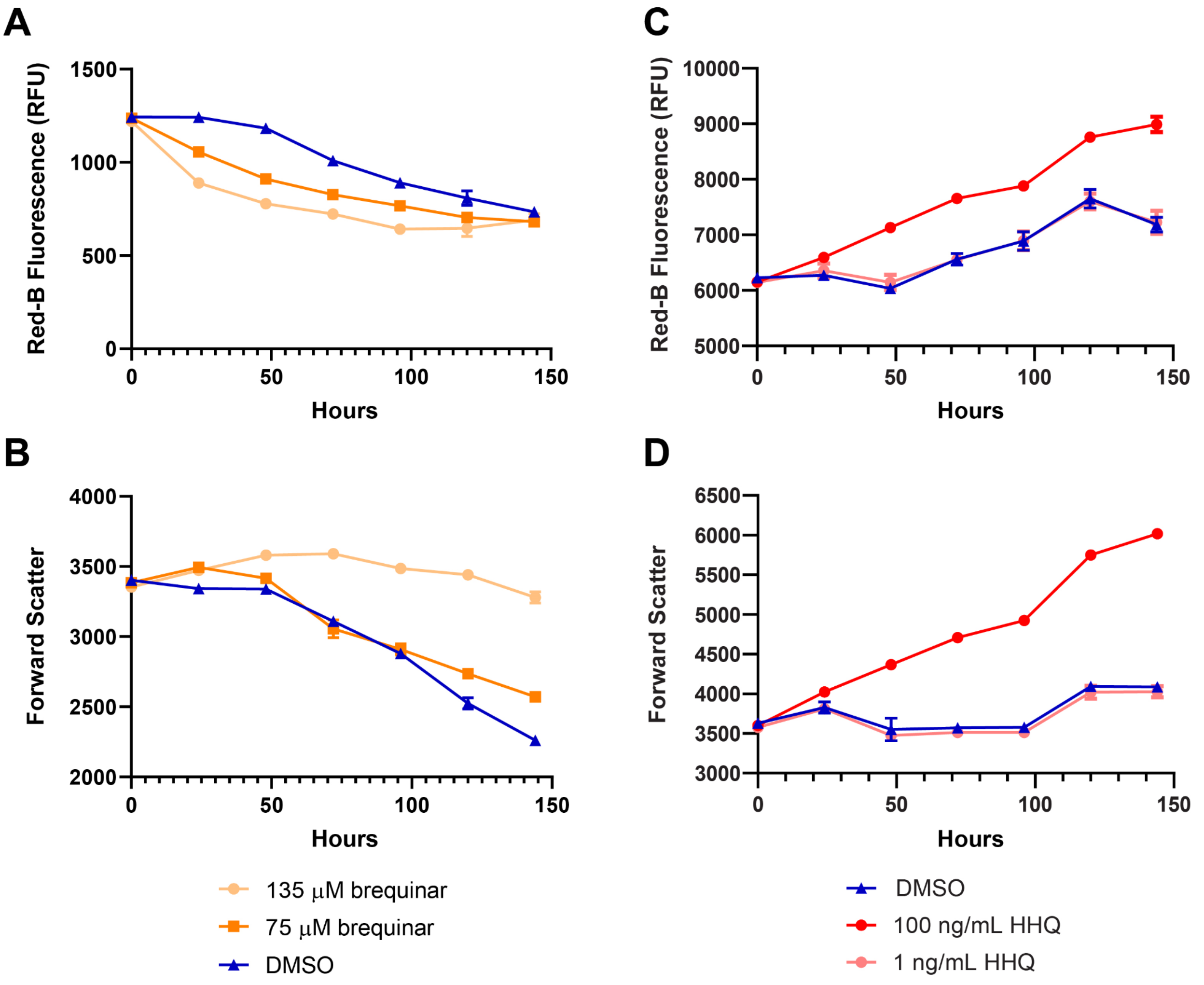

Supplemental Figure 7. Red-B fluorescence and forward scatter of brequinar-treated *E. huxleyi* 2090 cultures from Figure 4A compared to HHQ-treated *E. huxleyi* 2090 cultures from Pollara et al. 2021. *E. huxleyi* (CCMP2090) cultures were dosed with brequinar and monitored by flow cytometry for (**A**) red-b fluorescence (in relative fluorescence units) as a proxy for chlorophyll and (**B**) forward scatter as a proxy for cell size. For comparison, red-b fluorescence (**C**) and forward scatter (**D**) of HHQ-treated *E. huxleyi* (CCMP2090) obtained from Pollara et al. 2021 are also plotted. Means and standard deviations of biological triplicates are shown.

```
                       10        20        30        40        50
EhDHODH   1   ---------MLRRTASACAAGGVLAAGAYAAQGGDEGRRSVLLGLARADE
HsDHODH   1   MAWRHLKKRAQDAVIILGGGGLLFASYLMATGDERFYAEHLMPTLQGLLD
PtDHODH   1   MLHQAAQRVQRVVIRTAVGTTVVVGTVEWATHFPSQGR----------SS
DtDHODH   1   ----------MGVLAKLVGAAGAVGIGGFA-YYATAGA----------EQ

                       60        70        80        90       100
EhDHODH  42   QLRPALAALLPAEAFVALYSASRTPFLGALAASAGCGALPSAPS------
HsDHODH  51   PESAHRLAVRFTSLGLLPRARFQDSDMLEVRVLGHKFRNPVGIAAGFDKH
PtDHODH  41   QFYHDLVDHIVTPTMRRILDPETAHHAGIFFAEQGLSPR-FRPSALEQRC
DtDHODH  30   QFW--LISSM-GPAF-RLLDPETTHNLGIEAAKWGLFPKETRPDP----A

                      110       120       130       140       150
EhDHODH  85   -IGLRAMGLSFRNDLGNAAGLDKDGTLLDFNYALGAGYAVVGTVLSDPHS
HsDHODH 101   GEAVDGLYKMGFGFVEIGSVTPKPQEGNPRPRVFRLPEDQAVINRYGFNS
PtDHODH  90   VVSNSVFGKTFPNPIGLAAGFDKDGEVVQEMLDLGFGFVEIGTVTPQAQP
DtDHODH  72   ILRTRVWAKDFPNPIGLAAGFDKHAEAVEPLMKLGFGFVEIGSVTPRPQP

                      160       170       180       190       200
EhDHODH 135   GNLFDFLGGLWRGNAWTPLPSSGGALNSLGLPSKGVEPALRNIAAFRERH
HsDHODH 151   HGLSVVEHRLRARQQKQAKLTEDGLPLGVNLGKNKTSVDAAEDYAEGVRV
PtDHODH 140   GNPKP---RMFR------LVEDLGIINRYGFNSQGASTVEKNLKSFRSPQ
DtDHODH 122   GNPSP---RVFR------IKDLSATINRYGFNSLGADAVQDNLTLFAKKA

                      210       220       230       240       250
EhDHODH 185   GVPPQRAEHGR--------PAGDGGASFPVGVSIMGHPAHGDPADKLEGV
HsDHODH 201   LGPLADYLVVNVSSPNTAGLRSLQGKAELRRLLTKVLQERDGLRRVHRPA
PtDHODH 181   PIDSAKLSWPRYIWNFLYPPRHQSGL---VGVNIGKNKNSLDAIGDYVCN
DtDHODH 163   RED----------------PAAKPGL---LGVNLGKNKSSKDAATDYSIG

                      260       270       280       290       300
EhDHODH 227   LRCVRLCLPLADFIEINESCPNVHHGGHGGDATAELRKRLEAIVAERDA
HsDHODH 251   VLVKIAPDLTSQDKEDIASVVKELGIDGLIVTNTTVSRPAGLQGALRSET
PtDHODH 228   IRQLS---SLADYMVLNISSPNT--AGLRDLQQANSLRVLLTTCLTTRNE
DtDHODH 194   LTKLS---QHADYVVINISSPNT--PGLRALQGKKELEALVSQVKGTRDR

                      310       320       330       340       350
EhDHODH 277   AARGGGRRVPLLVKLG-DL--GEAEGTVRFLSRLGIDGVVALNTQKDYAS
HsDHODH 301   GGLSGKPLRDLSTQTIREMYALTQGRVPIIGVGGVSSGQDALEKIRAGAS
PtDHODH 273   M----THRVPLLVKLAPDLTDEELDQIANVCLEVGIDGIIITNTTNH---
DtDHODH 239   MRWGPSGPPPLVVKIAPDLTQEDKEDIAAVALKYKLDGLIVSNTTIT---

                      360       370       380       390       400
EhDHODH 324   FELPPADRALLDSYTARYGGGLSGPPILDRSSAQAAAAQAAVRELGLAGR
HsDHODH 351   LVQLYTALTFWGPPVVGKVKRELEALLKEQGFGGVTDAIGADHRR-----
PtDHODH 315   ---RPAD--LLSKHRG-EIGGLSGKPVKD----QSTECIRRLFRLT-DGK
DtDHODH 285   ---RPGA--VAQYPEGKEAGGLSGPPLMD----LATDVLADMYRLTGKGK

                      410       420       430       440       450
EhDHODH 374   FVVVHVGGVQSAADVQRSRATGAELRQWYTGLMHG----LARPEPDLL--
HsDHODH 395   --------------------------------------------------
PtDHODH 355   IPIIGVGGVGSGHDAYEKLKAGASLVQVYSMMVYQGPGVISRIRHDLATL
DtDHODH 327   IPLIGCGGVSNGEDAYRKIRAGASLVQLYTALAYAGPGVVPTIKAELARL

                      460       470       480       490       500
EhDHODH 417   -------------------------YERVTAPERHQRLRAAMR-------
HsDHODH 395   --------------------------------------------------
PtDHODH 405   MLENGQRSIVDVIGADHEDIFWRKREERIAQKRRRDTRISIETLPVKKTE
DtDHODH 377   LERDGFESVAAAVGVDHQP--GKRYENAGPQEGSRGTWWKQGS-------

EhDHODH 435   --
HsDHODH 395   --
PtDHODH 455   FA
DtDHODH 417   --
```

Supplemental Figure 8. Alignment of the predicted amino acid sequences for DHODH from *E. huxleyi*, human, and two additional phytoplankton species. Residues with at least 55% conservation are bolded, and 100% conservation are indicated with an asterisk. Highlighted in grey are alpha-helices predicted by PredictProtein (Bernhofer et al., 2021). Red indicates recombinant expression start sites. Residues found within 5 Å of HHQ in EhDHODH are marked orange, and those near a leflunomide derivative in HsDHODH in a reported crystal structure (PDB 31FQ) are in blue. EhDHODH, *Emiliania huxleyi*, R1EHA9; HsDHODH; *Homo sapiens*, Q02127; PtDHODH, *Phaeodactylum tricornutum*, XP 002183522.1; DtDHODH, *Dunaliella tertiolecta,* CAE0486972.1.

References

Baldwin, J., Farajallah, A. M., Malmquist, N. A., Rathod, P. K., & Phillips, M. A. (2002). Malarial dihydroorotate dehydrogenase. Substrate and inhibitor specificity. *The Journal of Biological Chemistry*, *277*(44), 41827–41834. https://doi.org/10.1074/jbc.M206854200

Bernhofer, M., Dallago, C., Karl, T., Satagopam, V., Heinzinger, M., Littmann, M., Olenyi, T., Qiu, J., Schütze, K., Yachdav, G., Ashkenazy, H., Ben-Tal, N., Bromberg, Y., Goldberg, T., Kajan, L., O'Donoghue, S., Sander, C., Schafferhans, A., Schlessinger, A., … Rost, B. (2021). PredictProtein—Predicting Protein Structure and Function for 29 Years. *Nucleic Acids Research*, *49*(W1), W535–W540. https://doi.org/10.1093/nar/gkab354

Eberhardt, J., Santos-Martins, D., Tillack, A. F., & Forli, S. (2021). AutoDock Vina 1.2.0: New Docking Methods, Expanded Force Field, and Python Bindings. *Journal of Chemical Information and Modeling*, *61*(8), 3891–3898. https://doi.org/10.1021/acs.jcim.1c00203

Garavito, M. F., Narvaez-Ortiz, H. Y., Pulido, D. C., Löffler, M., Judelson, H. S., Restrepo, S., & Zimmermann, B. H. (2019). Phytophthora infestans Dihydroorotate Dehydrogenase Is a Potential Target for Chemical Control – A Comparison With the Enzyme From Solanum tuberosum. *Frontiers in Microbiology*, *10*. https://www.frontiersin.org/articles/10.3389/fmicb.2019.01479

Jumper, J., Evans, R., Pritzel, A., Green, T., Figurnov, M., Ronneberger, O., Tunyasuvunakool, K., Bates, R., Žídek, A., Potapenko, A., Bridgland, A., Meyer, C., Kohl, S. A. A., Ballard, A. J., Cowie, A., Romera-Paredes, B., Nikolov, S., Jain, R., Adler, J., … Hassabis, D. (2021). Highly accurate protein structure prediction with AlphaFold. *Nature*, *596*(7873), Article 7873. https://doi.org/10.1038/s41586-021-03819-2

Pettersen, E. F., Goddard, T. D., Huang, C. C., Meng, E. C., Couch, G. S., Croll, T. I., Morris, J. H., & Ferrin, T. E. (2021). UCSF ChimeraX: Structure visualization for researchers, educators, and developers. *Protein Science: A Publication of the Protein Society*, *30*(1), 70–82. https://doi.org/10.1002/pro.3943

Pollara, S. B., Becker, J. W., Nunn, B. L., Boiteau, R., Repeta, D., Mudge, M. C., Downing, G., Chase, D., Harvey, E. L., & Whalen, K. E. (2021). Bacterial Quorum-Sensing Signal Arrests Phytoplankton Cell Division and Impacts Virus-Induced Mortality. *MSphere*, *6*(3), e00009-21. https://doi.org/10.1128/mSphere.00009-21

Trott, O., & Olson, A. J. (2010). AutoDock Vina: Improving the speed and accuracy of docking with a new scoring function, efficient optimization and multithreading. *Journal of Computational Chemistry*, *31*(2), 455–461. https://doi.org/10.1002/jcc.21334
